# Understanding the impact of external context on community-based implementation of an evidence-based HIV risk reduction intervention

**DOI:** 10.1186/s12913-017-2791-1

**Published:** 2018-01-09

**Authors:** Alison B. Hamilton, Brian S. Mittman, Danielle Campbell, Craig Hutchinson, Honghu Liu, Nicholas J. Moss, Gail E. Wyatt

**Affiliations:** 1 UCLA Department of Psychiatry and Biobehavioral Sciences, 760 Westwood Plaza, 38-240 NPI, Box 175919, Los Angeles, CA 90024-1759 USA; 2 VA Center for the Study of Healthcare Innovation, Implementation, & Policy, VA Greater Los Angeles Healthcare System, 11301 Wilshire Boulevard, Los Angeles, CA 90073 USA; 3 Kaiser Permanente Southern California, Department of Research and Evaluation, 100 S. Los Robles Avenue, Pasadena, CA 91101-2453 USA; 4 UCLA Department of Medicine, 760 Westwood Plaza, Los Angeles, CA 90024-1759 USA; 5 HIV STD Section, Alameda County Public Health Department, 1000 Broadway, Suite 310, Oakland, CA 94607 USA

**Keywords:** Implementation, External context, Community-engaged research, Hybrid, HIV prevention, Policy

## Abstract

**Background:**

Organizational context plays a critical role in the implementation of evidence-based interventions. Implementation research to date has focused largely on internal, rather than external, context. This paper presents key features of external context and their impact on implementation of Eban II, an evidence-based HIV/AIDS prevention intervention currently being tested in community-based organizations (CBOs). We examine external context factors that have influenced implementation, highlighting the ways in which client needs, agency resources, and changing policies permeate the theorized boundary between internal and external context, affecting both organizational capacity for implementation research and implementation processes themselves.

**Methods:**

Staff (*n* = 91) across participating CBOs completed a baseline survey of organizational functioning; a subset of key stakeholders (*n* = 15) completed semi-structured interviews. Client participants (*n* = 84) completed a baseline survey. Process notes and organizational documents were also analyzed.

**Results:**

Organizational readiness for implementation was high across the organizations. However, despite apparent readiness, external contextual barriers to implementation were substantial. Three categories of barriers were identified: (1) client needs as a manifestation of social determinants of poverty, (2) community agency resources, and (3) local and national policy changes. Clients’ psychosocial vulnerability affected their everyday lives and priorities, which thereby affected the regularity and intensity of their interface with CBOs, and hence their participation in our intervention. CBOs typically lacked staffing and space. Furthermore, changing federal and state policy priorities destabilized the CBOs, which had a ripple effect on our study. Drawing on community-engaged research principles, we made numerous adjustments to the intervention format and structure according to the preferences and contexts of the CBOs. Had we not adjusted to external contextual factors, the organizations would not have been able to maintain their involvement and provide the intervention to their clients, despite expressed, genuine commitment to shared goals.

**Conclusions:**

Community-based implementation studies need to address complex organizational and client needs, using community-engaged research principles. If these studies are community-based among vulnerable populations, they need to more thoroughly evaluate, monitor, and address the ways in which external contextual factors impinge upon implementation processes and outcomes, with a parallel need for more comprehensive measures of fiscal, political, and social determinants of implementation success.

**Trial registration number:**

NCT01829282 (Registered April 11, 2013).

## Background

Implementation science facilitates the integration of HIV/AIDS research, practice, and policy [1] by looking beyond individual risk behaviors to the context of healthcare services, to understand and improve care for those with pronounced healthcare needs. The broader social, political, and economic contexts of risk [2] surround and influence agencies that are providing services to HIV-positive individuals, which means that interventions for this population need to be tailored locally and contextually. [3]

Extensive evidence confirms that organizational context plays a critical role in the implementation of evidence-based interventions. [4] To date, most efforts to measure contextual factors in implementation science have focused on “internal context,” including organizational settings, cost effectiveness, staff readiness, training, technical assistance, information technology, and sustainability. [5, 6] Less empirical attention has been paid to “external context,” i.e., the broader environment in which organizations are situated. Numerous frameworks provide guidance for conceptualizing factors that exert influences on organizations, [7, 8] but very little research has been conducted on these factors. [9] The widely used Consolidated Framework for Implementation Research (CFIR) [10] characterizes “outer context” as inclusive of patient needs and resources (i.e., extent to which patient needs, as well as barriers and facilitators to meet those needs, are accurately known and prioritized by the organization), cosmopolitanism (i.e., degree to which an organization is externally networked with other external organizations), peer pressure (i.e., pressure to implement an intervention), and external policy and incentives. Despite these and other conceptualizations of external context, few studies articulate the ways in which external context affect the course of implementation in real time.

This paper presents key features of external context and their impact on implementation of Eban II, an evidence-based HIV/AIDS prevention intervention currently being tested in community-based organizations (CBOs) across Northern and Southern California. The eight-year Eban I study (“Eban” is a Yoruba term for “fence”) was designed to reduce risk of HIV transmission among heterosexual, serodiscordant African American couples across multiple settings in the United States; results confirmed the intervention’s efficacy in reducing HIV risk. [11] The Eban II study was funded by the National Institutes of Mental Health as a Type II hybrid effectiveness-implementation study [12] that builds upon prior research to examine the intervention’s effectiveness in real-world community-based organizations (CBOs). [13] Eban II also, by necessity, has evolved as a community-engaged research study [14] that has had to accommodate and address HIV service community needs and vulnerabilities.

Here, we examine external context factors that have influenced the progress and success of implementation of this evidence-based intervention, and we highlight the need to further develop our understanding and measurement of the ways in which external context can affect implementation. We emphasize the fluid nature of the fiscal, political, and social environment in which CBOs operate. Specifically, over the last 10 years, the landscape of HIV research has changed and continues to change significantly in ways that impact delivery and uptake of evidence-based interventions. Some of these changes include flattening of budgets, shifting policy priorities, gentrifying neighborhoods in which CBOs deliver services and in which impacted communities live, and changing organizational expectations. We highlight the ways in which policies, patient needs, and other aspects of the external context domain permeate the theorized boundary between internal and external context, affecting both organizational capacity for implementation research and implementation processes themselves.

## Materials and methods

### Organizational contexts and overview of study design

Community-based organizations (CBOs; *n* = 10) in Los Angeles and Alameda Counties agreed to serve as sites for this study. At the planning stage of the study (prior to funding), all of the CBOs were well-established, serving large numbers of HIV-infected African Americans. These CBOs expressed particular interest in offering services to couples because they did not provide such services prior to the funding of the study. These agencies were identified as having met seven key elements identified in the literature [15] as important in determining agencies’ readiness to implement a new intervention: (1) a respected local community advocate; (2) strong administrative support; (3) formal organizational commitments and stability; (4) commitment of necessary resources to incorporate the intervention into existing services; (5) intervention credibility within the community; (6) adequate facilitators/staff; and (7) potential for the intervention to be self-sustaining or willing to seek additional funding.

### Overview of methods

At baseline (pre-implementation), a mixed methods evaluation was conducted using survey and qualitative methods (semi-structured interviews and analysis of process notes and organizational documents). Staff at the participating organizations completed the baseline evaluation within the same timeframe in order to have a quantitative assessment of organizational readiness across CBOs at one point in time.

### Procedures: Organizations & Staff

Subsequent to a study “kick-off” (i.e., a week-long orientation and training of the site coordinators, facilitators, agency administrators and staff, and data collectors), baseline evaluation data collection activities commenced at the 10 participating CBOs. Lists of agency staff and their email addresses were compiled by the site coordinators, and invitation emails were sent, each with a personalized, secure link to the baseline web-based survey (described below). A consent script was provided at the beginning of the survey, and initiation of the survey constituted consent to participate. Those who completed the survey were included in a raffle for a video streaming device. All study procedures were approved by the University of California Los Angeles Institutional Review Board.

Of the 10 participating CBOs, five had agreed to serve as initial implementation sites, i.e., to offer the Eban intervention. Qualitative interviews were conducted at these five sites with key stakeholders who were integral to implementation. Key stakeholders (*n* = 16; approximately 3 per initial implementation site) were contacted via email or phone and invited to participate in an interview. Those who agreed to participate (all but one who had her deputy complete the interview due to time constraints) completed written informed consent and then completed the semi-structured interview (described below) either in person or over the phone. All interviews but one were recorded and transcribed. No compensation was provided for completing the interview.

Process notes, taken by several members of the research team, include minutes of all study-related calls and meetings as well as field notes recorded during the kick-off meetings and several site visits. Organizational documents (e.g., mission statements, annual reports) were gathered from each participating CBO. In addition, a key implementation strategy [16] throughout the course of the study was monthly interagency calls, during which implementation challenges were raised by representatives from the participating CBOs.

### Sample: Staff

Staff eligible to complete the baseline survey were those who had a role in providing or managing direct care for clients (*n* = 101; approximately 10 per agency). Agency administrators and/or site coordinators provided the research team with lists of eligible staff, who were recruited via email. A total of 91 staff members (90%) completed the survey.

Key stakeholders, i.e., a subset of staff and administrators directly involved in Eban implementation at the initial implementation sites, were eligible for the qualitative interview based on their roles as agency administrators, site coordinators, and intervention facilitators.

### Measures: Staff

The electronic Staff Survey captured basic demographics of staff including education level and professional experience. Subscales from the Texas Christian University Survey of Organizational Functioning (TCU SOF) [5] assessed motivational factors, program resources, staff attributes, and organizational readiness. All subscales of the SOF have demonstrated good internal consistency and validity. Of particular relevance to this analysis, two items in the survey pertain to factors outside the organization (i.e., “Current pressures to make program changes come from…” “funding and oversight agencies,” and “accreditation or licensing authorities”). Response categories are 1 = Strongly Disagree to 5 = Strongly Agree.

The key stakeholder interview guide contained questions about understanding of study goals, expectations for implementation, experience with couples-based interventions and with the target population, anticipated challenges with the intervention (including recruitment and logistics), potential facilitators of implementation, perceived benefits of the intervention, participation in and value of network calls, perceptions of research team support, anticipated sustainability of the intervention, and suggestions for improving implementation.

The following organizational features were gathered from agency reports and verified by administrators: number of staff (full- and part-time), number of clients served, past-year turnover of non-clerical staff, and any notable funding changes (loss or gain of funding, private or public).

### Data analysis: Staff survey

To analyze the SOF, subscale scores were obtained by summing responses to the items (reversing scores when necessary), dividing the sum by number of items included (yielding an average), and multiplying by 10 in order to rescale final scores so they ranged from 10 to 50. Means for each scale were examined across sites and in comparison to normative data. In addition, 25th and 75th percentiles were calculated for further comparisons. Subscale scores above 30 indicate areas of strength, or “readiness” in that the respondents generally agree that the organization has the attribute in a given subscale. Scores below 30 (which are unusual) indicate areas of weakness that might need attention prior to change efforts. The standard deviations (SDs) of the scores indicate the level of consensus on any given subscale; SDs above 9 indicate considerable variability in responses and prompt questions about why the subscale topic is perceived differently across respondents within a given organization.

### Data analysis: Key stakeholder interviews

Transcripts of key stakeholder interviews, process notes, and archival material were analyzed by the research team using directed content analysis [17] to identify theorized aspects of external context affecting implementation. Specifically, the analysis team, led by the lead author, used targeted coding procedures (facilitated by ATLAS.ti, a qualitative data analysis software package) to identify narrative and/or notes pertaining to the CFIR external context domains. This approach is consistent with other teams that have used CFIR domains to reduce and organize qualitative data. [18]

### Procedures: Clients

Following baseline evaluation data collection, we used community outreach and marketing to recruit and screen African-American, HIV-serodiscordant heterosexual couples (see [13] for detailed eligibility criteria). All participants were asked to provide written verification of their HIV and STI status at screening. If they were unaware of their status, they had to obtain documented verification prior to enrolling. They also had to obtain HIV and STI screens at three and six months post-enrollment. All individuals completed written informed consent, and then completed a computerized baseline survey comprised of demographic questions as well as several standardized measures (see below) [13].

### Measures: Clients

Demographic characteristics of participating clients included gender, age, education, marital/relationship status, income, ethnicity, health insurance status, and verified HIV-serostatus and STI status that were tested within the past 30 days. Substance use/abuse (frequency and amount of alcohol use each day in the past 3 months and age of first use) was measured by the CAGE [19] to assess dependence on alcohol, and section B of the NIDA Risk Behavior Assessment (RBA) [20] to assess the frequency, modality, and level of use of licit and illicit drugs in the past month.

### Data analysis: Client survey

Frequencies, cross-tabulations, and chi-square were calculated for the demographic characteristics and substance use/abuse.

## Results

### Organizational functioning

Overall, organizational readiness was high across the organizations. The strongest characteristics across agencies were Adaptability (M = 39.1; SD = 6.0), e.g., “You are willing to try new ideas even if some staff members are reluctant.”; Growth (M = 37.1; SD = 7.1), e.g., “This program encourages and supports professional growth.”; and Organizational Mission (M = 36.4; SD = 7.3), e.g., “This program operates with clear goals and objectives.” The Pressures for Change subscale pertaining in part to external context showed little variation across organizations, with a range in mean scores from a low of 31.0 (SD = 11.3) to a high of 35.7 (SD = 7.6).

### External context

Our analysis of key stakeholder interviews, process notes, organizational documents, and client demographic measures revealed that external contextual barriers to implementation in Eban II cluster in three categories: (1) community agency resources (aligned with CFIR’s cosmopolitanism and peer pressure constructs), (2) patient needs as a manifestation of social determinants of poverty (i.e., CFIR’s patient needs and resources construct), and (3) local and national policy changes (i.e., CFIR’s external policy and incentives construct).

#### Community agency resources

Implementation barriers were encountered at the agency level and across agencies, which were highly networked with one another (in some cases, sharing staff and space). During pre-implementation, 10 agencies made a commitment to receive training in the Eban intervention and to serve as intervention sites. Kick-off meetings were held in the two geographic regions (Northern and Southern California) in which the directors, site coordinators, and facilitators were trained to deliver Eban as designed and approved by NIH. These kick-off meetings served as important venues for “peer pressure” across the agencies: agency representatives were pleased to see one another at these meetings and described being “proud” that they would be delivering the intervention. However, once the agencies came to a full understanding of their roles and responsibilities as active sites, five agencies requested to change their participation as active sites and decided to become “referral sites,” citing a lack of staff time and/or space to successfully implement the intervention; subsequently an additional agency decided that they were not able to offer the intervention due to diminishing staff.

Barriers at the remaining agencies included 1) staff turnover and limited space; 2) incentives for participation; and 3) transportation. Even though these barriers occurred at the agency level, they were also shared across agencies, and problems were often attempted to be solved through the networks that these agencies had with one another.

##### Barrier 1: Staffing and space

The four participating sites experienced their own staffing and space issues as they began to roll out the Eban intervention. Staffing issues included a high turnover rate and overall reduced staffing as budgets were reduced due to changes in the national and state funding environment, leading to fewer billable hours. Also, a unique feature of Eban II is the modeling of healthy male-female interactions by male-female dyad co-facilitators during each session. Although all sites were initially enthusiastic about the value of this approach, only two sites had male staff members available to take on the facilitator role. Another site hired a new part-time staff member specifically for this role and the male Study Coordinator in Oakland took on this additional responsibility until a “floating” substitute male facilitator could be hired. Available staff time to deliver the intervention as designed also had to be adapted to adjust to the real-world circumstances of high turnover, limited budgets, and staff availability. For example, an adjustment was made to allow one facilitator to conduct the couple sessions without a co-facilitator when necessary. In some instances, sites shared staff in order to meet the needs of the study. We interpreted this as an example of the “cosmopolitanism” construct, whereby the network among the agencies facilitated implementation that was otherwise difficult for each agency on its own.

##### Barrier #2: Incentives

Adjustments to the incentive schedule also had to be made to accommodate the expectations of the Eban participants. Once we were in the field, we quickly learned that multiple non-research HIV care and prevention interventions in these settings rely on incentives (including food) to drive engagement in services. Most of the Eban participants had previously participated in research studies at the agencies before and expected to be paid for their time. However, we were restricted to providing incentives only for research-related activities (i.e., providing HIV/STI documentation and completing the computerized patient interview at three different time points) due to grant reviewer stipulations that paying participants would not be “real world” implementation. Participants were expected to attend the eight-week intervention sessions without payment as if the intervention were a regular part of their services at the site. However, to achieve regular attendance to these sessions, incentives had to be provided. Furthermore, as noted below, the participants were low-income and often unstably housed, therefore food and transportation also had to be provided to encourage their active participation during each session. To accommodate the need to incentivize the Eban sessions, the incentive schedule was rearranged to spread the reimbursement payments throughout the participation period and provide a small cash payment ($10) for each session attended as scheduled.

##### Barrier #3: Transportation

Another logistical barrier that became apparent early during implementation was the location of the intervention sites in the cities of Los Angeles and Oakland. Few of the intervention participants had access to cars and using public transportation on a fixed income could become expensive. To adjust for this, bus tokens and rail passes were provided to enrolled participants. In addition, in some cases, participants attended the intervention sessions across different participating agencies (depending on where they could travel to with time and funds available), again reflecting the influence of cosmopolitanism on community-based implementation. Transportation also posed a barrier to obtaining the required HIV/STI documentation, as facilities that conducted testing were often not convenient to the participating agencies (discussed further below).

#### Patient needs

Social determinants of poverty include low income/unemployment, homelessness, substance abuse, and chronic co-morbidities. Patients’ psychosocial needs had a substantial effect on their utilization of services. Notably, the participating CBOs were mainly focused on HIV-positive individuals and had interest in, but little history of, meeting the typically substantial needs of their clients’ HIV-negative partners.

We observed and substantiated considerable vulnerability among the HIV-positive and HIV-negative clients who consented to participate in this study (Table 1). The vast majority (90%) were unemployed at baseline; 95% were making $1650 or less per month. Almost three-quarters (71%) had a history of incarceration; over one-third (37%) had been in inpatient substance abuse treatment. In terms of substance abuse, over one-third (39%) reported that in the past three months, they felt they should cut down on drinking; HIV-negative participants were significantly more likely to endorse this item. HIV-negative participants were also significantly more likely to not have a regular doctor.

**Table 1:** Client Characteristics

Characteristic	HIV-negative (*n* = 42)	HIV-positive (n = 42)	Total (*n* = 84)
	n or mean (SD)	n or mean (SD)	n or mean (SD)
Age (years)	51.43 (10.10)	48.83 (8.11)	50.13 (9.20)
Education			
Less than HS	7	11	18
HS diploma/GED	25	20	45
College (some and completed)	10	11	21
Employment Status			
Unemployed	36	40	76
Household Income (~$1335 FPL)			
< $400/month	10	7	17
$400–$850/month	18	15	33
$851–$1650/month	13	17	30
Unmarried	29	32	61
Number Dependent Children	.81 (1.86)	.78 (1.837)	.80 (1.84)
Living Situation			
Own home/apt	19	28	47
In family’s home/apt	3	2	5
In partner’s home/apt	12	3	15
Other (someone else’s home/apt, rooming house, welfare-type place, group home/institution, on the street)	7	9	16
History of Incarceration	29	31	60
Spent Time in Inpatient Drug Treatment	12	19	31
Overall quality of life			
Excellent	2	8	10
Very good	8	10	18
Good	17	14	31
Fair	14	8	22
Poor	1	2	3
Self-reported hepatitis-C	7	6	13
Had drink, past month	26	18	44
How often drink, past month			
Not at all/a few times	7	11	18
A few times each week	16	5	21
Every day	3	2	5
Past 3 months, feel you should cut down on drinking*	21	12	33
Past 3 months, annoyed by people who criticize drinking	7	6	13
Past 3 months, feel bad/guilty about drinking	12	5	17
Past 3 months, have drink in morning for hangover*	8	2	10
Past 3 months, used substances to get high/relax	23	21	44
Past 3 months, number of times sniff/snort heroin	4.04 (18.75)	4.29 (19.64)	4.16 (18.95)
Past 3 months, number of times smoked marijuana	19.48 (27.36)	12.48 (22.96)	16.14 (25.31)
Past 3 months, number of times used illegal drugs	7.59 (15.73)	4.90 (12.32)	6.28 (14.06)
Past 3 months, number of times injected drugs	2.82 (12.78)	4.9 (19.64)	3.53 (16.31)
Past year, used larger amounts or for longer periods	8	9	17
Past year, tried to cut down but couldn’t	9	13	22
Past year, a lot of times involved with drugs	9	5	14
Past year, got so high couldn’t work/caused accident	5	4	9
Past year, spent less time working because of drugs	4	3	7
Past year, drugs caused emotional/psychological problems	10	5	15
Past year, drugs caused problems with others	9	4	13
Past years, drugs caused problems with physical health	7	3	10
Past year, had to increase amount of drug	6	4	10
Past year, needed care but couldn’t get it	3	4	7
Don’t have regular doctor*	10	2	12

*p < 0.05

Patients’ health and life circumstances greatly impacted regular attendance at the Eban sessions (one per week for eight weeks), with several participants experiencing tumult during the course of participation. Consistent attendance was impacted by criminal justice involvement (arrest, incarceration), hospitalizations, loss of housing, and loss of employment. For example, one participant was arrested during the first Eban session and his conviction meant that he could no longer participate in the study. His partner agreed to continue coming without him, however with her primary source support now incarcerated, she relapsed and did not complete all eight sessions. Other participants were hospitalized during participation, leading to a longer than expected lapse of time to complete follow-up interviews and HIV/STI testing. Housing status and employment also played a role in attendance. One of the male participants only came to sessions when he was unemployed and needed the additional income. When he found work, he broke up with his partner and declined to finish the intervention. He later returned when he lost work and once again became unemployed. Another couple was not able to secure stable housing and moved two times during the eight-week intervention leading to absences and brief periods of the dissolution of the relationship.

#### Policy

Several policies have posed barriers to implementation, including policy related to HIV/STI testing, and funding for HIV treatment and prevention.

##### HIV/STI testing

At the onset of the study in 2011, there was inconsistent information about how available STI testing would be in the community. Whereas HIV testing was and is free and readily available, securing free STI testing for chlamydia, gonorrhea, and syphilis was more challenging. According to the study design, participants were asked to confirm their HIV and STI status at baseline and again at eight weeks and three months. Further, each agency had a limited budget for repeat HIV/STI testing within the study time frame, with the state funding repeat tests every six months. There was little flexibility in the standard wait time of six months for research participants to obtain repeat testing. Furthermore, STI testing was often not available within the Eban sites, requiring participants to travel significant distances to receive their results; also, there was often a lengthy delay between testing and receiving results. These factors influenced participants’ willingness to receive an STI test and contributed to delayed entry into and nonoptimal retention in the study.

##### Treatment as prevention

During the study, we observed and heard from our participating CBOs that state and federal funding for HIV prevention interventions was reduced or redirected to emphasize biomedical interventions and HIV risk among men who have sex with men. These changes disrupted long-standing HIV outreach, education and testing interventions the study sites had historically delivered to the study population.

## Discussion

**Fig. 1:**
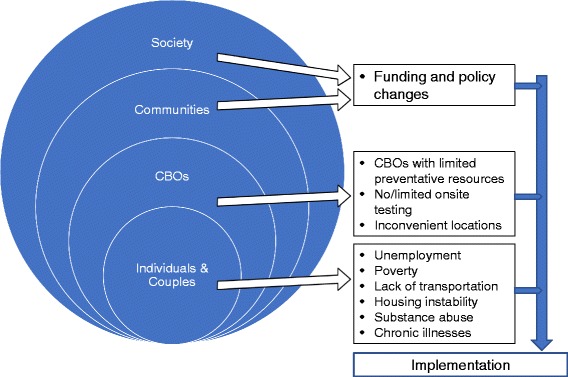
Interactive barriers to Eban II implementation across multiple socio-ecological levels [2]

The external context barriers facing community-based implementation trials are unique and differ substantially from the neatly defined characteristics of clinical trials. In this study we found that—despite high internal organizational readiness for implementation, interest in providing services to couples, and commitment to the intervention—patient needs, community agency resources, and local and national policy changes have directly impacted implementation processes (see Fig. 1). Patient needs were pronounced and affected the consistency of their utilization of services. The couples participating in Eban represent a different population from those typically served by our participating agencies, i.e., HIV-service organization tended to focus on HIV-positive individuals, not couples nor HIV-negative partners. We observed and substantiated considerable vulnerability among the HIV-positive and HIV-negative clients who consented to participate in this study. This vulnerability affected clients’ everyday lives and priorities, which thereby affected the regularity and intensity of their interface with service organizations, and hence their participation in our intervention which was situated within the organizations.

Our participating organizations appeared to be “ready” for implementation, yet community agency resources were typically lacking with regard to staffing and space. We surmise that the degree of cosmopolitanism among the participating agencies in some ways facilitated implementation, in that staff and space was often shared across agencies in order to make the intervention accessible to the clients. Furthermore, participating agencies frequently had opportunities that provided their clients with engagement incentives (e.g., for participation in clinical trials) that extended well beyond the budgetary parameters of our implementation study. Thus our study, with minimal incentives available, often fell short of clients’ expectations for and experiences with research participation. Other studies have similarly found that incentive schedules need to be changed during the course of implementation in the “real world.” [21] Furthermore, community organizations did not provide the STI testing resources that were required for participation in our study. This was a gap in our team’s understanding of these resources, which then affected our participants’ ability to comply with the study protocol. Given that syphilis is at epidemic proportions in some populations using PReP (e.g., men who have sex with men) within LA and Alameda County, [22] more effort needs to be devoted to ensuring availability and consistency of testing, as well as concerted follow-up educational efforts related to risk reduction practices.

This study has notable limitations. First, we did not use CFIR to design the study; instead, we applied this framework analytically because of our consistent field-based observations about the impact of external context. This may have limited the depth and specificity of our information about the CFIR external context domains because they were not “built in” to our data collection instruments. Second, we are only able to speak to the influence of external context on the agencies that ultimately participated by delivering the intervention; because six agencies did not deliver the intervention, they also did not participate in data collection subsequent to the baseline organizational survey, so we are unable to provide empirical evidence about the extent to which external context factors affected their lack of participation in implementation.

This study has brought to light the many challenges associated with community-based implementation of an evidence-based intervention that was previously tested under rigorous conditions. This hybrid implementation/effectiveness trial is substantially affected by the external context of the participating organizations and the clients they serve. We found that changing priorities at the federal and state levels destabilized community agencies, [23] which had a ripple effect on our study. Our study was not the highest priority for the agencies nor the clients, despite strong organizational interest in providing novel and evidence-based services to couples, and openness to the intervention among clients. In order to accommodate the needs of our participating organizations, we had to make numerous adjustments to the intervention format and structure according to the preferences and contexts of the CBOs (expressed during regular implementation calls and site visits). [21] Had we not drawn on community-engaged research principles during the course of implementation, we believe that the organizations would not have been able to maintain their involvement in the study and provide the intervention to their clients, despite expressed and genuine commitment to shared goals.

## Conclusions

The face-value appeal of an intervention can easily be overridden by survival needs at both the organizational and client levels. Therefore, in conclusion, we pose several suggestions for community-based implementation studies: 1) they need funds for compensation that are consistent with community-based expectations; 2) they need to address complex organizational and client needs, using community-engaged research principles [24, 25]; 3) if they are community-based among vulnerable populations, they need to more thoroughly evaluate, monitor, and address the ways in which external contextual factors impinge upon implementation processes and outcomes, with a parallel need for more comprehensive measures of fiscal, political, and social determinants of implementation success.
